# Repellent effects of the essential oils of *Cymbopogon citratus* and *Tagetes minuta* on the sandfly, *Phlebotomus duboscqi*

**DOI:** 10.1186/s13104-017-2396-0

**Published:** 2017-02-15

**Authors:** Albert Kimutai, Moses Ngeiywa, Margaret Mulaa, Peter G. N. Njagi, Johnstone Ingonga, Lydia B. Nyamwamu, Cyprian Ombati, Philip Ngumbi

**Affiliations:** 1 Department of Biological Sciences, University of Kabianga, P.O. Box 2030-20200, Kericho, Kenya; 2 University of Eldoret, P.O. Box 1125-30100, Eldoret, Kenya; 3 Center for Biotechnology Research and Development, Kenya Medical Research Institute, P.O. BOX 54840 - 00200, Mbagathi Rd., Nairobi, Kenya; 4 Moi University, P.O. Box 3900-30100, Eldoret, Kenya

**Keywords:** *Phlebotomus duboscqi*, Lemon grass, *Cymbopogon citratus*, Mexican marigold, *Tagetes minuta* L., Essential oil, Repellent

## Abstract

**Background:**

The sandfly, *Phlebotomus duboscqi* is a vector of zoonotic cutaneous leishmaniasis (ZCL) that is an important public health problem in Eastern Africa. Repellents have been used for protection of humans against vectors of ZCL and other vectors that transmit killer diseases including malaria, Rift Valley fever, dengue, and yellow fever. The repellent effects of different doses of the essential oils from the lemon grass, *Cymbopogon citratus* and Mexican marigold, *Tagetes minuta* were evaluated in a two-chamber bioassay against 3- to 7-day-old unfed females of *P. duboscqi* in the laboratory. The results were compared with those that were obtained when test animals were treated with an equivalent dose of diethyl-3-methylbenzamide, which is a repellent that is commonly used as a positive control.

**Results:**

Overall, percentage repellency increased with increasing doses of the essential oils while biting rates decreased with increasing concentrations of the oils. Further, the oil of *C. citratus* was more potent than that of *T. minuta* with regard to protection time and biting deterrence. The effective doses at 50% (ED_50_) and at 90% (ED_90_) for the oil of *C. citratus,* were 0.04 and 0.79 mg/ml, respectively. Those of the oil of *T. minuta* were 0.10 and 12.58 mg/ml. In addition, the percentage repellency of 1 mg/ml of the essential oils of *C. citratus* and *T. minuta* against sandflies was 100% and 88.89%, respectively. A lower dose of 0.5 mg/ml of the oils, elicited 89.13% repellency for *C. citratus* and 52.22% for *T. minuta.*

**Conclusion:**

The laboratory tests showed that the essential oils of the two plants were highly repellent to adult sand flies, *P. duboscqi*. Thus, the two essential oils are candidate natural repellents that can be used against *P. duboscqi* due to their high efficacy at very low doses, hence, the envisaged safety in their use over chemical repellents. It remains to carry out clinical studies on human subjects with appropriate formulations of the oils prior to recommending their adoption for use against the sandflies.

## Background

Phlebotomine sandflies transmit leishmaniases which is a group of diseases that to date puts at risk of disease 350 million people in 88 countries worldwide [1]. The World Health Organization [2] estimates that over 2.3 million new cases of leishmaniasis occur each year and that at least 12 million people are presently infected worldwide. In some countries, sandflies also carry and transmit other zoonoses such as bartonellosis [3], phleboviruses [4, 5], certain flaviviruses, orbiviruses and vesiculoviruses [6, 7], that cause health problems for humans and domestic animals.

In Kenya, phlebotomine sandflies transmit visceral and cutaneous leishmaniases. Visceral leishmaniasis (VL), caused by *Leishmania donovani* is transmitted by *Phlebotomus martini* (Diptera: Psychodidae) [8, 9]. On the other hand, *P. duboscqi* sandflies transmit *L. major,* one of the causative agents of zoonotic cutaneous leishmaniasis (ZCL) [5, 10]. The current management strategy for leishmaniasis is mainly based on chemotherapy for treatment of infected cases and use of insecticides in vector control to reduce transmission [11, 12]. However, usage of highly persistent and toxic synthetic insecticides has led to development of resistance in vector populations. In addition, environmental pollution due to the repeated applications is a challenge. Thus, the harmful side effects of these chemicals on both animals and humans have progressively limited their usage and have led to increased interest in alternative new natural chemicals that are environmentally safe, affordable and effective in management of leishmaniases. In this context, screening of natural products has received the attention of researchers around the world. Since many diseases that are transmitted by insects such as malaria, dengue, yellow fever, leishmaniasis and Chaga’s disease are endemic in developing countries, the search for insecticides and repellents of botanical origin has been driven by the need to find new products that are effective, but also safer and more affordable than currently available products [13]. In recent decades, research on the interactions between plants and insects has revealed the potential use of plant metabolites or allelochemicals for this purpose [14]. Some chemical constituents of essential oils from various plants have insecticidal properties [15]. Specific compounds isolated from plant extracts or essential oils have been tested for fumigation purposes [16].

Essential oils of an appreciable number of plants have been shown to be repellent against various haematophagous arthropods [17–19]. Lemongrass, *Cymbopogon* spp. produce the most used natural repellents in the world [20]. For example, essential oils from *Cymbopogon martinii* elicited 100% repellency against *Anopheles* sp. mosquitoes in field tests for 12 h [21]. Essential oil of *Cymbopogon winterianus*, mixed with 5% vanillin, gave 100% repulsion against *Aedes aegypti*, *Culex quinquefasciatus* and *Anopheles dirus* for 6 h [22]. Lemongrass, *C. citratus* essential oil is obtained from the aerial parts of the plant. The plant has been widely recognized for its enthnobotanical and medicinal usefulness [23]. Other documented effects of essential oils of plants include insecticidal [24–29] antifungal [30], antimicrobial [31, 32], and the therapeutic properties [23]. However, there are relatively few studies that have been carried out to determine the efficacy of essential oils from citronella as arthropod repellents [33] and specifically against sandflies.

On the other hand, the essential oil of the Mexican marigold, *T. minuta*, has been shown to have both larvicidal and adulticidal effects on mosquitoes [34–36]. The active components were isolated from different parts of the plant. Green et al. [34], reported mosquito larvicidal activity in the extract of *Tagetes minuta* flowers. Perich et al. [36] compared biocidal effects of the whole-plant extracts of three *Tagetes* spp. and showed that *T. minuta* had the greatest biocidal effect on the larvae and adults of *Ae. aegypti* (L.) and *Anopheles stephensi* (L). Bioassays carried out with simultaneous steam distillates of *T. minuta* flowers showed 90% larval mortality at lethal concentrations (LC_90_) of 4 and 8 ppm and against the adult at 0.4 and 0.45% against *Aedes aegypti* and *Anopheles stephensi*, respectively [36]. Recently, Ireri et al. [37] demonstrated that methanol and ethyl acetate crude extracts of *T. minuta* derived from the aerial parts had significant mortality against both male and female *P. duboscqi*, Neveu Lemaire (Diptera: Psychodidae). Further, Mong’are et al. [38] found that similar crude extracts reduced the fecundity of *P. duboscqi* by 53%.

The present study sought to evaluate the repellent and insecticidal effects of the essential oils of the lemon grass, *C. citratus* and *T. minuta* against adult sandflies, *P. duboscqi*.

## Methods

### Extraction of essential oils

Extraction of essential oils of *T. minuta* and lemon grass, *C. citratus* and gas chromatography–mass spectrometric (GC–MS) analysis were done in the Behavioural and Chemical Ecology Department (BCED) laboratory at the International Centre for Insect Physiology and Ecology (*icipe*), Kasarani, Nairobi, Kenya. Repellency experiments were conducted at the Centre for Biotechnology Research and Development (CBRD) of the Kenya Medical Research Institute (KEMRI), Nairobi in the Leishmaniasis laboratory. The institute has a viable sandfly colony and the requisite facilities that facilitated the studies to be undertaken.

### Collection of plant materials

Fresh leaves of the lemon grass, *Cymbopogon citratus* were collected from the equatorial rainforest in Kakamega, Kenya. A voucher specimen reference number CBRD/CC/001/2015 was deposited at KEMRI’s Center for Biotechnology Research and Development (CBRD) for future reference. The leaves were screened and dry and/or damaged ones were discarded. The remaining good leaves were used for extraction while still fresh. On the other hand, floral and foliar parts of *T. minuta* plants were collected from Marigat division of Baringo district, Rift Valley province, Kenya. The plants’ identities were confirmed by a taxonomist at the University of Nairobi. These were packed in a cold box and transported to the International Centre for Insect Physiology and Ecology (*icipe*), Kasarani, Nairobi, Kenya where extraction of the essential oils was done. A voucher specimen reference number CBRD/TM/001/2015 was deposited at KEMRI’s Center for Biotechnology Research and Development (CBRD) for future reference.

### Extraction of essential oils of *Tagetes minuta* and *Cymbopogon citratus*

Extraction of the essential oil of the lemon grass *c. citratus* was done as described by Adeniran and Fabiyi [39]. The fresh leaves were immersed in distilled water after which they were subjected to steam distillation. The mixture of steam and the volatile oil generated was passed through a condenser and collected in a flask. Then, a separating funnel was used to separate the oil from water. The recovered oil was dried using anhydrous sodium sulphate and kept in a refrigerator at 4 °C for subsequent use [39].

For the extraction of the essential oil from *T. minuta*, fresh plant material was sliced and hydro-distilled by using a Clevenger-type apparatus [40], with slight modifications [41]. Heat was provided by a heating-mantle equipped with a thermostat and the temperature maintained at 90 °C. The plant material was immersed in distilled water then placed into a 2 litre round-bottomed flask and hydro-distilled for 2 h. The distillate was collected as the essential oil band above the water [42].

### Sand fly colony

Sandflies were obtained from a colony of *P. duboscqi* Neveu Lemaire that originated from Marigat Division, Baringo district, Rift Valley, and were maintained at the Centre for Biotechnology Research and Development (CBRD) insectaries in Kenya Medical Research Institute, Nairobi. The colony of *P. duboscqi* was established using field-captured females that were held in cages and maintained according to the methods of Beach et al. [43] with some modifications. Briefly, female sandflies were fed on blood using Syrian golden hamsters that had been anaesthetized with sodium pentobarbitone (Sagatal^®^). The hamsters’ underbellies were usually shaven using an electric shaver for easy access for feeding by sandfly. The sandflies were reared at 28 ± 1 °C, and an average RH of 85–95% and 12:12 h (light: dark) photoperiod in Perspex^®^ insect rearing cages. Sandflies were fed ad libitum on slices of apple that were supplied daily as a source of carbohydrates.

### Preparation of oil extracts for repellent tests

Test samples of the essential oils of *T. minuta* and *C. citratus* were prepared by reconstituting measured amounts of the essential oils in olive oil to have a series of concentrations of 0.125; 0.250; 0.500; 0.750 and 1 mg/ml. Separate experiments using different cages were done in triplicates and hamsters were treated with the above preparations of the essential oils To prevent any cross-over effects between treatments with the different concentrations, each test with a given dose of each oil was applied to one hamster per cage. The olive oil and a standard repellent, *N*,*N*-diethyl-3-methylbenzamide (DEET) were used as negative and positive controls, respectively.

### Assessing repellent effects of essential oils

Sandflies, *Phlebotomus duboscqi* were obtained from a colony which was maintained at the CBRD insectary. The basic design of this experiment was a modification of the World Health Organization WHO Pesticide Evaluation Scheme (WHOPES) [44]. Experiments were carried out in the laboratory within tunnels constructed from glass cages with plaster of Paris on their bases. Two such cages, each measuring 25 cm (width) × 25 cm (height) x 40 cm (length), were joined on their open ends with an adhesive tape to form a tunnel measuring 25 × 25 × 80 cm. Before joining the two cages with tapping material, a removable cardboard frame of 1 cm thick that had holes (of 20 mm diameter) drilled through was fitted in between the cages.

The repellency tests were conducted as previously described by Kasili et al. [45], with some modifications. In the shorter section of the tunnel, a restrained hamster, anesthetized with sodium pentobarbitone (Sagatal^®^), and acting as a bait (host) was placed. Separate experiments using different cages were conducted in which hamsters were treated by smearing their legs, tail, and mouth parts with 0.1 ml of the various serial concentrations of 0.125; 0.250; 0.500; 0.750 and 1 mg/ml of *T. minuta* and *C. citratus* extracts.

**Fig. 1:**
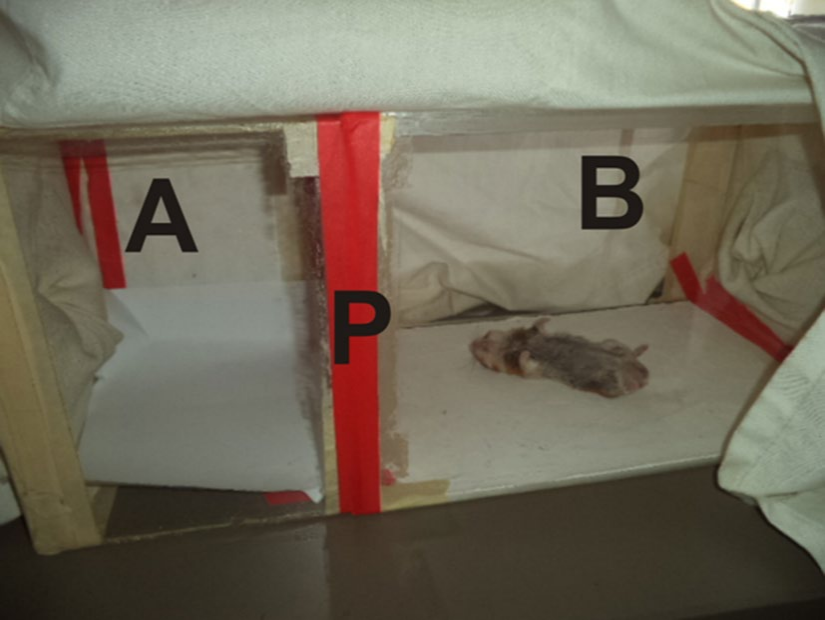
Perspex cage measuring 25 X 25 x 80 cm (length) separated by a removable cardboard frame (P). The restrained hamster smeared with the oil preparation on the legs, tail, and mouth parts is on the test chamber B. Sand flies were held in chamber A and had to make contact with the removable cardboard and pass through the perforated partition (P) to reach the bait (hamster) in chamber B. Repellency was determined by counting the number of sand flies that landed on the hamsters’ legs, tail, and mouth parts for a period of five minutes each at intervals of 30 min

**Fig. 2:**
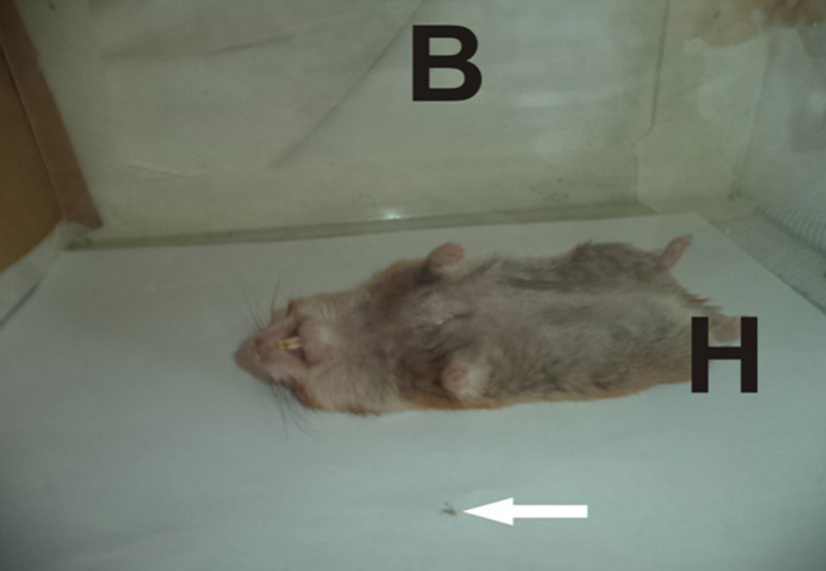
Shows the restrained hamster (H) smeared with the oil preparation on the legs, tail, and mouth parts on the test chamber (B). A sand fly that had crossed into the chamber is shown at the tip of the arrow

On one side of the flight tunnel were 100 sandflies that were held in Perspex cage while on the other, a hamster that had been smeared with the oil preparation on the legs, tail, and mouth parts was placed. Sand flies that were pre-starved for 4 h or more prior to testing were used for the experiment. Different concentrations of the oils were tested in different cages and each was replicated three times. Each test cage contained 100 flies, thus for a given dose, 300 flies were used. In addition, for each dose, only one hamster was used. The bioassay set up was such that flies flew freely in in the tunnel but had to make contact with the removable cardboard and pass through the holes to reach the bait (hamster) (Figs. 1, 2).

Following the release of flies in the tunnel, landing counts (the number of sand flies that landed on the hamsters’ legs, tail, and mouth parts that had been smeared with oil preparations) was done for five minutes each at intervals of 30 min between 08:00 and 11:00 h. Mean percent repellency for each concentration was calculated based on the data of the three replicates at the given times of observation. Percent repellency for the test oils and DEET was calculated using the formula:

$$ {\text{Repellency}}\;(\% ) = \left( {{\text{N}}{-}{\text{R}}} \right)/{\text{N}} \times 100 $$

where, N = number of flies landing on the negative control side; R = number of flies landing on side treated with test oil or DEET. Thus, efficacy of the candidate repellent could be assessed relative to DEET.

During tests, the bioassay room was maintained at 27 °C and 80–95% RH. To obtain an acceptable estimate of effective dose (ED), ED_50_ and ED_90_, the treated areas on the hamster were swabbed with isopropanol pads.

### Estimation of Protection time

To determine protection time, a modified screened two-cage arena [46] was used. For the tests, 100 nulliparous, 5–7 day old sandflies, *P. duboscqi* were released into the holding cage. The sandflies were free to fly in the tunnel, make contact with the piece of removable perforated cardboard partition, pass through the holes and locate the restrained hamster in the adjacent cage. As in the other bioassays, the hamsters were treated by smearing their legs, tail, and mouth parts with 0.1 ml of concentrations of 0.125; 0.250; 0.500; 0.750 and 1 mg/ml of *T. minuta* and *C. citratus* extracts. Following the release of flies into the tunnel, their biting ability was monitored between 08:00 to 11:00 h at intervals of 30 min. Observations were done for three minutes within each half hour and the total number of sandflies biting on the treated and control areas recorded. If no observations were made for the first 3 min of every half an hour exposure, the experiment was discontinued until the next half hour. The test was continued until at least two bites occurred and were followed by a confirmatory bite (second bite) in the subsequent exposure period. The time between application of the test oil and the second successive bite was recorded as the protection time.

### Data analysis

All experiments were replicated three times. Data on repellency, protection time, and biting rates were recorded using the Microsoft Excel programme. Control groups in the experimental bioassays with >20% repellency were repeated. Where repellency in the control groups fell between 5 and 20%, the observed percentage repellency was corrected using Abbott’s formula [47]. The dose-repellency data was analysed by log-probit method of Finney [48] and effective concentrations for 50% (ED_50_) and 90% (ED_90_) repellency determined. Statistical significance of the recorded repellency of the various test concentrations and the controls were analyzed using one-way analysis of variance (ANOVA) at *P* < 0.05.

## Results

This study sought to determine the repellent activity of two essential oils of the lemon grass, *C. citratus* and *T. minuta*. The results of GC–MS of *C. citratus* and *T. minuta* demonstrated that the oils were dominated by monoterpene hydrocarbons. The monoterpene fraction for *C. citratus* was characterized by a high percentage of geranial (20.45%), myrcene (14.24%), neral (11.57%), and verbenene (9.26%) while that of *T. minuta* composed of dihydro-tagetone (21.15%), (E)-tagetone (16.21%), (Z)-tagetone (14.99%), (Z)-β-ocimene (9.84%), limonene (7.40%), allo-ocimene (6.69%) and (Z)-ocimenone (4.12%). In the dose–response study for determining effective dose, the results on ED_50_ and ED_90_ values are shown in Table 1. The ED_50_ and ED_90_ values of essential oil of lemon grass, *C. citratus* were determined to be 0.04 and 0.79 mg/ml, respectively, while those for the oil of *T. minuta* were 0.1 and 12.58 mg/ml, respectively. In addition, the percentage repellency of the two essential oils against *P. duboscqi* is presented in Table 2. The essential oil of *C. citratus* at three concentrations (1, 0.75 and 0.5 mg/ml) provided the highest repellency with 100%, 87.67 and 89.13 resp1ectively at 180 min. On the other hand, the repellency of *T. minuta* essential oil at similar concentrations of 1, 0.75 and 0.5 mg/ml was relatively lower than that of *C. citratus* at 88.89%, 79.56 and 52.2 respectively at 180 min. The most potent oil was that of *C. citratus* at 1 mg/ml that elicited an average repellency of 99.8% (range 99.8–100%; ED_50_ 0.04) and a mean biting rate of 0.8 at various concentrations (Table 3).

**Table 1:** Effectiveness of *C. citratus* and *T. minuta* essential oils against *Phlebotomus duboscqi* tested on hamsters as repellents

Repellents	No. flies	ED50 (mg/ml)	95% CL^a^ (mg/ml)	ED90 (mg/ml)	95% CL^a^ (mg/ml)
*C. Citratus*	100	0.039	0.039 ± 0.0082	0.79	0.79 ± 0.0082
*T. minuta*	100	0.10	0.1 ± 0.0367	12.58	12.58 ± 0.0367
Controls					
DEET 0.196 mg/ml	100	0.0009	0.0009 ± 0.0001	0. 0015	0. 0015 ± 0.497

ED50: Effective dose that causes 50% of prohibiting of bites; ED90: Effective dose that causes 90% of prohibiting of bites

^a^Mean dosages are significantly different (*P* < 0.05) from each other if 95% confidence limits (CL) do not overlap

**Table 2:** Repellent activities (%) of Lemon grass and *T. minuta* essential oils in five concentrations ranging from 0.125 to 1 mg/ml against *P. duboscqi*, Neveu Lemaire

	Oil conc. (mg/ml)	Repellency (%)					
		30 min	60 min	90 min	120 min	150 min	180 min
Essential oil	0.125	96.875 ± 0.20	84.62 ± 2.08	53.40 ± 2.14	52.05 ± 0.38	43.91 ± 0.19	51.3 ± 1.71
	0.25	96.875 ± 0.19	98.21 ± 0.33	98.81 ± 0.19	90.66 ± 0.51	83.57 ± 1.90	59.13 ± 0.51
*C. citratus*	0.50	97.81 ± 0.30	97.69 ± 0.20	98.09 ± 0.20	92.94 ± 0.20	93.70 ± 1.00	89.13 ± 1.20
	0.75	99.38 ± 0.20	97.44 ± 0.19	98.34 ± 0.19	99.0 ± 0.19	100.0 ± 0.00	87.67 ± 0.67
	1.00	100.00 ± 0.00	100.00 ± 0.00	98.72 ± 0.20	99.77 ± 0.20	100.00 ± 0.00	100.0 ± 0.00
	0.125	83.72 ± 1.00	58.46 ± 2.14	48.94 ± 2.83	44.31 ± 3.42	28.69 ± 2.36	21.49 ± 2.27
*Tagetes minuta*	0.25	90.23 ± 0.80	64.10 ± 2.20	70.21 ± 2.08	60.98 ± 4.55	51.3 ± 3.01	46.81 ± 5.81
	0.50	96.88 ± 0.33	91.53 ± 0.33	86.53 ± 0.67	74.51 ± 3.84	58.22 ± 3.34	52.22 ± 7.78
	0.75	98.14 ± 0.40	100.00 ± 0.00	97.87 ± 0.19	88.45 ± 0.84	64.35 ± 2.34	75.96 ± 1.76
	1.00	100.0 ± 0.00	100.00 ± 0.00	98.81 ± 0.19	98.68 ± 0.00	91.30 ± 0.60	88.89 ± 1.57
Controls							
DEET	0.196	100.0 ± 0.00	100.0 ± 0.00	100.0 ± 0.00	100.0 ± 0.00	100.0 ± 0.00	95 ± 1.70

**Table 3:** Biting rates of *P. duboscqi* sandflies when tested against five ranging from 0.125 mg/ml to 1.0 mg/ml of *C. citratus* and *T. minuta* essential oils DEET and Tween 80

Essential oil	Oil conc. (mg/ml)	Mean number of biting sandflies					
		30 min	60 min	90 min	120 min	150 min	180 min
*C. citratus*	0.125	0.6 ± 0.20	6.0 ± 2.08	21.90 ± 2.14	21.1 ± 0.38	25.8 ± 0.19	22.4 ± 1.71
	0.25	1.0 ± 0.19	0.67 ± 0.33	0.56 ± 0.19	4.11 ± 0.51	7.56 ± 1.90	18.8 ± 0.51
	0.5	0.7 ± 0.30	0.9 ± 0.20	0.9 ± 0.20	0.7 ± 0.30	2.9 ± 1.00	5.0 ± 1.20
	0.75	0.20 ± 0.20	0.89 ± 0.19	0.78 ± 0.19	0.00 ± 0.00	0.0 ± 0.00	0.00 ± 0.00
	1	0.00 ± 0.00	0.00 ± 0.00	0.60 ± 0.20	0.1 ± 0.01	0.00 ± 0.00	0.00 ± 0.00
	0.125	0.22 ± 0.19	16.2 ± 2.14	23.8 ± 2.83	28.4 ± 3.42	32.8 ± 2.36	36.9 ± 2.27
*Tagetes minuta*	0.25	0.78 ± 0.19	14.22 ± 2.22	14.0 ± 2.08	19.89 ± 4.55	22.44 ± 3.01	25.0 ± 5.81
	0.5	0.8 ± 0.20	0.2 ± 0.20	6.3 ± 0.67	13.1 ± 3.84	19.22 ± 3.34	22.44 ± 7.78
	0.75	0.78 ± 0.40	0.00 ± 0.00	0.78 ± 0.19	0.33 ± 0.33	16.4 ± 2.34	11.3 ± 1.76
	1	0.00 ± 0.00	0.00 ± 0.00	0.56 ± 0.19	0.67 ± 0.00	0.11 ± 0.20	5.22 ± 1.57
Controls	0.196 mg/ml	0.00 ± 0.00	0.00 ± 0.00	0.00 ± 0.00	0.00 ± 0.00	0.00 ± 0.00	0.33 ± 0.13
DEET Tween 80		43.00 ± 6.30	39.00 ± 2.50	47.00 ± 7.1	51.00 ± 5.5	46 ± 1.00	47.00 ± 7.50

DEET—*n*,*n*-diethyl-3-methylbenzamide

Tween 80—polysorbate 80

In general, the percentage repellency of the two essential oils increased when the concentration of these essential oils increased, in contrast, biting rates decreased when the concentration increased. The results showed significant differences in both the percentage of repellency and the number of sand flies biting (P < 0.05).

Data on the protection time conferred by different concentrations of the two essential oils are shown in Table 4. The data shows that 1 mg/ml and 0.75 of essential oil of *C. citratus provided* 100% protection for up to 3 h. On the other hand, 1 mg/ml of the oil of *T. minuta* conferred lesser protection as compared to that of *C. citratus* for up to 150 min.

**Table 4:** Protection time of Lemon grass and *T. minuta* essential oils in five concentrations ranging from 0.125 to 1.0 mg/ml against *P. duboscqi*, Neveu Lemaire

Essential oils			Controls	
Oil conc. (mg/ml)	Protection time (min)		Protection time (min)	
	Lemon grass	*T. minuta*	DEET	Tween 80
0.125	60	30	>180	<30
0.25	120	30		
0.5	120	60		
0.75	180	120		
1	180	150		

## Discussion

The essential oils of *C. citratus* and *T. minuta* have not previously been tested against the sandfly *P. duboscqi*. However, most of the previous studies on repellency by essential oil of the lemon grass were carried out with mosquitoes. Other plant-derived compounds that have been shown to reduce mosquito and/or sandfly trap catches include geraniol, linalool, and citronella [49]. The results of this study demonstrate high repellent effects of these essential oils on the adults of the sandfly, *P. duboscqi*. Grasses of *Cymbopogon* spp. have been traditionally used for repelling mosquitoes in jungle regions such as the Bolivian Amazon [50]. Plants of this genus produce the most used natural repellents in the world [51]. A wide range of extracts and essential oils isolated from these plants have been tested against a broad range of species of arthropods. In particular, formulations of the oil of *C. citratus* in paraffin oil have been successfully utilized [52]. However, the essential oil of *Cymbopogon nardus* oil that was evaluated against *Cydia pomonella* (Lepidoptera: Tortricidae) was inactive [53]. Further, oils of *C. nardus* and *Cymbopogon flexuosus* were ineffective on the cigarette beetle, *Lasioderma serricorne* (Coleoptera: Anobiidae) [52].

In one particular study, in which the ED_50_ was closest to what was obtained for this study, Phasomkusolsil and Soonwera [27] demonstrated that the essential oils of various species of plants including *C. citratus, Cymbopogon nardus, Syzygium aromaticum* and *Ocimum basilicum* exhibited high repellency against *Ae. aegypti* with the ED_50_ at less than 0.045 mg/cm^2^ of the substrate. In the same study, oils of *C. citratus, C. nardus* and *S. aromaticum* showed repellency against *An. dirus* with ED_50_ at less than 0.07 mg/cm^2^. On the other hand, the essential oils of *C. citratus, C. nardus, S. aromaticum, O. basilicum* and *Cananga odorata* gave strong effective dose (ED_50_) values of less than 0.003 mg/cm^2^ of substrate when tested against *Culex quinquefasciatus* [27]. Similar findings have been documented by Soonwera and Sinthusiri [54] who obtained 87.9% effective repellency of the essential oil of *C. citratus* among other oils tested on the house fly, *Musca domestica*.

Dried plants of *T. minuta* are usually indoors to repel a broad range of insect species [37, 55]. Repellent activity of *Tagetes* species have been reported against *Anopheles gambiae*, the vector of malaria [55]. *Tagetes* species have also showed insecticidal activity against stored product pests [56]. The efficacy of 100 ppm of *T. minuta* essential oil against head lice *Pediculus humanus capitis* (Phthiraptera: Pediculidae) was evaluated and it was found to be toxic to the insects [56]. The toxic effect of the oil of *T. minuta* to dipterans was attributed to the presence of terpenes [35]. In addition, the GC–MS results obtained in this study indicated that the *T. minuta* essential oil prepared in that study included β-ocimene that has been shown to be a tick repellent [57]. The soft tick, *Hyalomma rufipes* adults showed a significant dose-repellent response to the essential oil *T. minuta* [42].

With regard to protection time of *C. citratus* essential oil, a similar study [27] using the method of inserting a human arm treated with 0.21 mg/cm^2^ of essential oil into a cage with mosquitoes. They found that the oil gave the longest protection periods against three mosquito species; 72 min for *Ae. aegypti*, 132 min for *An. dirus* and 84 min for *Cx. quinquefasciatus*. However, the essential oils *C. nardus* and *Syzygium aromaticum*, exhibited moderate repellency against *Ae. aegypti, An. dirus* and *Cx. quinquefasciatus.*

In a study carried out in Ethiopia, *Phlebotomus bergeroti* Parrot adults that are vectors of visceral leishmaniasis were repelled by neem (*Azadirachta indica*) and chinaberry (*Melea azedarach*) oils at 2 and 5% formulations in coconut oil. These oil formulations provided protection of up to 98.3% protection for up to 9 h at the higher concentration, under laboratory conditions. In tests against field populations of *Phlebotomus orientalis* Parrot and *P. bergeroti*, 2 and 5% neem oil in coconut oil mixtures and DEET, the essential oil and DEET were not effective. Other essential oils that have been tested include garlic clove (*Allium sativum*) oil for which 1% preparation elicited a repellency of 97.0% mature female sandflies, *Phlebotomus papatasi* Scopoli [58].

Over the years, researchers have demonstrated that effectiveness of repellents over several hours can be improved by synergizing the repellent with a base or fixative materials such as vanillin, salicylic acid and mustard and coconut oils, among others [22, 59, 60]. However, the effectiveness of the repellents depends on multiple factors including the type of repellents (active ingredients), formulation, mode of application, environmental factors (temperature, humidity, and wind), the attractiveness of individual people to insects, loss due to removal by perspiration and abrasion, the sensitivity of the insects to repellents, and the biting density [61–67].

## Conclusion

In conclusion, the results of this study show that the essential oils of *C. citratus and T. minuta* at relatively physiological doses have strong repellency against adult sandflies, *P. duboscqi*. It remains to carry out chemical analytical studies to characterize the candidate repellent compounds by testing them in bioassays individually and in blends.

## Abbreviations

ANOVA: analysis of variance
CBRD: Centre for Biotechnology Research and Development
DEET: diethyl-3-methylbenzamid
ED_50_: effective dose 50
ED_90_: effective dose 90
GC–MS: gas chromatography–mass spectrometry
KEMRI: Kenya Medical Research Institute
VL: visceral leishmaniasis
ZCL: zoonotic cutaneous leishmaniasis
